# Comparative analysis of starch structure and multi-omics profiles reveals candidate pathways associated with a novel soft-waxy maize trait

**DOI:** 10.3389/fpls.2026.1784081

**Published:** 2026-04-10

**Authors:** Dongpu Ji, Yi He, Junhao Ran, Ziyi Fu, Xuanyu Liu, Zhiqiang He, Guangtong Xing, Jiuguang Wang, Lian Zhou

**Affiliations:** 1 Maize Research Institute, College of Agronomy and Biotechnology, Southwest University, Beibei, Chongqing, China; 2 Department of Plant Science, College of Agriculture, Missouri State University, Springfield, MO, United States

**Keywords:** eating quality, maize, metabolomic, multiscale properties, starch structure, transcriptomic

## Abstract

A novel soft-waxy maize, characterized by soft texture and slow retrogradation after cooking, was identified in this study. To investigate the structural and molecular features associated with this distinct trait, we conducted a comprehensive comparative study integrating analyses of starch physicochemical properties, molecular structure, transcriptomics and metabolomics. Soft-waxy maize starch showed a lower amylose content, reduced swelling power, distinct pasting characteristics (including a higher peak viscosity time and lower system viscosities), and higher gelatinization temperatures compared to waxy maize starch. Molecular structure characterization indicated a higher Mw and Mn, lower polydispersity, and an altered amylopectin chain-length profile, with increased proportions of B1 chains and decreased proportions of A and B2 chains. Transcriptome profiling identified differential expression of key starch biosynthesis genes, with *SSIIIb-2* emerging as a candidate gene potentially contributing to the observed structural variations. Integrated metabolomic and transcriptomic profiling revealed shifts in metabolic pathways that may be associated with the observed textural and palatability traits. These results provide a multi-scale characterization of a new type of maize with unique sensory profile, which offer a foundation for developing comprehensive evaluation methods and suggests potential targets for breeding waxy maize with enhanced palatability.

## 1 Introduction

As the predominant component of global starch production, maize (*Zea mays* L.) starch serves as a vital food resource for both humans and animals and a key raw material for diverse industrial applications. Maize starch consists of two glucose polymers: amylose and amylopectin. Structurally, amylose is primarily a linear chain composed of α-1,4-linked glucose, while amylopectin is a highly branched molecule due to the presence of α-1,6 linkages (Bertoft et al., 2012). Generally, in normal maize starch, amylose accounts for 25–28% of the composition, whereas in waxy maize starch, the amylose content is markedly lower, ranging from 0% to 8% (Bajaj et al., 2018). Therefore, waxy maize starch is relatively rich in amylopectin, contributing to its superior pasting properties and corresponding broad application potential, particularly in adhesives (Sarka and Dvoracek, 2017). Additionally, compared to normal and sweet maize starch, waxy maize starch exhibits the highest degree of crystallinity, system viscosity, and gelatinization temperatures (Yu et al., 2015).

Maize starch biosynthesis is coordinated and controlled by four series of enzymes: ADP-glucose pyrophosphorylase (AGPase), starch synthase (SS), starch branching enzyme (SBE), and starch debranching enzyme (DBE) (James et al., 2003; Tetlow et al., 2004; Hennen-Bierwagen et al., 2009). AGPase catalyzes the condensation of glucose-1-phosphate (G1P) to produce ADP-glucose (ADPG), a key precursor that provides glucose residues for the synthesis of both amylose and amylopectin. AGPase functions as a heterotetramer composed of large (LSU) and small (SSU) subunits. SSs then elongate glucan chains: granule-bound starch synthase (GBSS) is responsible for amylose synthesis (Delrue et al., 1992; Hu et al., 2022), while soluble starch synthases (SSI, SSII, SSIII) primarily elongate amylopectin chains with varying degrees of polymerization (DP) (Ball and Morell, 2003; Jeon et al., 2010; Keeling and Myers, 2010; Liu et al., 2015). SBEs introduce α-1,6-glycosidic linkages to create branched structures (Zeeman et al., 2010), and DBEs (isoamylase and pullulanase) prune improperly positioned branches to refine amylopectin architecture (Zeeman et al., 2010). Mutations in these genes produce well-characterized starch phenotypes. For instance, endosperm of the *sbeIIb* (*ae*) mutant maize contains over 60% amylose (Li et al., 2008), while mutations in *sugaryl* (*sul*), encoding ISAI, result in elevated sucrose levels, reduced amylopectin content, and decreased starch branching in maize kernels (James et al., 1995). Moreover, various mutations in genes involved in starch biosynthesis can differentially influence starch development and accumulation, leading to alterations in starch structure and functional properties (Tziotis et al., 2005; Li et al., 2010; Zhang et al., 2019).

The functional properties of starch, which are fundamentally governed by its structural characteristics, play a decisive role in the eating quality and processing suitability of starch-based products. Palatability, as a key functional property, is characterized by a set of distinct attributes, including roughness, stickiness, cohesiveness, hardness, and chewiness. Consumer preference strongly favors a soft, cohesive, and non-gritty texture, traits largely governed by the structural and functional properties of endosperm starch (Bett-Garber et al., 2001). For instance, soft rice, widely favored in Asia, is characterized precisely by low hardness and high stickiness (Nakamura et al., 2016; Wangcharoen et al., 2016). Research indicates that starch structure directly influences these properties: soft rice starch exhibits lower pasting temperature, final and setback viscosity, along with a higher proportion of short side chains in amylopectin (Li et al., 2016; Nakamura et al., 2021; Wang et al., 2020). Recent study indicates that metabolomics combined with transcriptomic data may help to clarify the metabolic and genetic basis of waxy corn flavor (Luo et al., 2024).

In our breeding program for fresh-eating maize, we identified a novel waxy maize inbred line exhibiting remarkably soft texture and slow retrogradation after cooking. We designated this unique germplasm as “soft-waxy maize”. This germplasm represents a valuable genetic resource for improving eating quality in waxy corn varieties, which can inform the development of various maize products. This raises a key question: what distinguishes soft-waxy maize from conventional waxy maize at the structural, functional, and molecular levels? To address this question, we compared the starch structural characteristics, functional properties, and the associated genetic and metabolic mechanisms between the two inbred lines. Specifically, in the present study, we aimed to: evaluate its physicochemical, such as pasting, and thermal properties; characterize the fine molecular structure, including chain-length distribution and molecular weight; and integrated metabolomic and transcriptomic approaches to identify candidate genes and pathways potentially involved in determining the soft-waxy phenotype. These findings are expected to provide a foundation for further processing of this novel maize with a unique taste, and may offer insights into the starch biosynthesis mechanism to facilitate maize breeding of special starch types for various applications.

## 2 Materials and methods

### 2.1 Plant materials

Five waxy maize inbred lines (designated 1–5) commonly utilized in the Chongqing maize breeding programs, were obtained from Southwest University to collectively represent the range of phenotypic variation in eating quality within our breeding germplasm. All inbred lines originated from the same founder parent combination and were developed through successive generations of self-pollination using the pedigree breeding method. The inbred lines were obtained through continuous selection for the target trait, and they are expected to possess a high degree of genetic similarity. These inbred lines were subjected to sensory evaluation by an expert tasting group, and the resulting scores are presented in **Table 1**.

**Table 1:** Sensory profiles of five cooked maize inbred lines.

Inbred line no.	Color	Flavor	Stickiness	Hardness	Roughness
1	8.7 ± 0.4^ab^	5.7 ± 0.5^c^	7.1 ± 0.4^a^	6.7 ± 0.3^a^	7.2 ± 0.4^a^
2	8.0 ± 0.5^b^	6.5 ± 0.4^bc^	6.9 ± 0.3^ab^	5.0 ± 0.4^b^	6.7 ± 0.3^a^
3	9.1 ± 0.3^a^	7.3 ± 0.4^a^	7.0 ± 0.3^a^	4.5 ± 0.3^b^	5.0 ± 0.4^b^
4	5.9 ± 0.4^c^	6.4 ± 0.5^bc^	6.4 ± 0.4^b^	6.2 ± 0.4^a^	6.0 ± 0.4^ab^
5	8.3 ± 0.4^ab^	7.0 ± 0.4^ab^	6.3 ± 0.4^b^	3.3 ± 0.3^c^	3.8 ± 0.5^c^

Values represent mean ± SD from three replicate sessions (n = 6). Different letters within the same column indicate significant differences (*P* < 0.05, LSD test).

Panel composition: A trained sensory panel consisting of 6 members from the Maize Research Institute, Southwest University, participated in the evaluation. All panel members had prior experience in sensory evaluation of maize products.
Sample preparation: Kernels from each inbred line were harvested at fresh-eating stage [25 days after pollination (DAP)], immediately husked, steamed for 25 minutes, and cooled to 50 °C.
Evaluation procedure: Kernels from each inbred line were presented in random order in coded containers. Each inbred line was evaluated in triplicate across three separate sessions. Five sensory attributes were evaluated using a 10-point linear intensity scale (0 = low, 10 = high).

**Figure 1:**
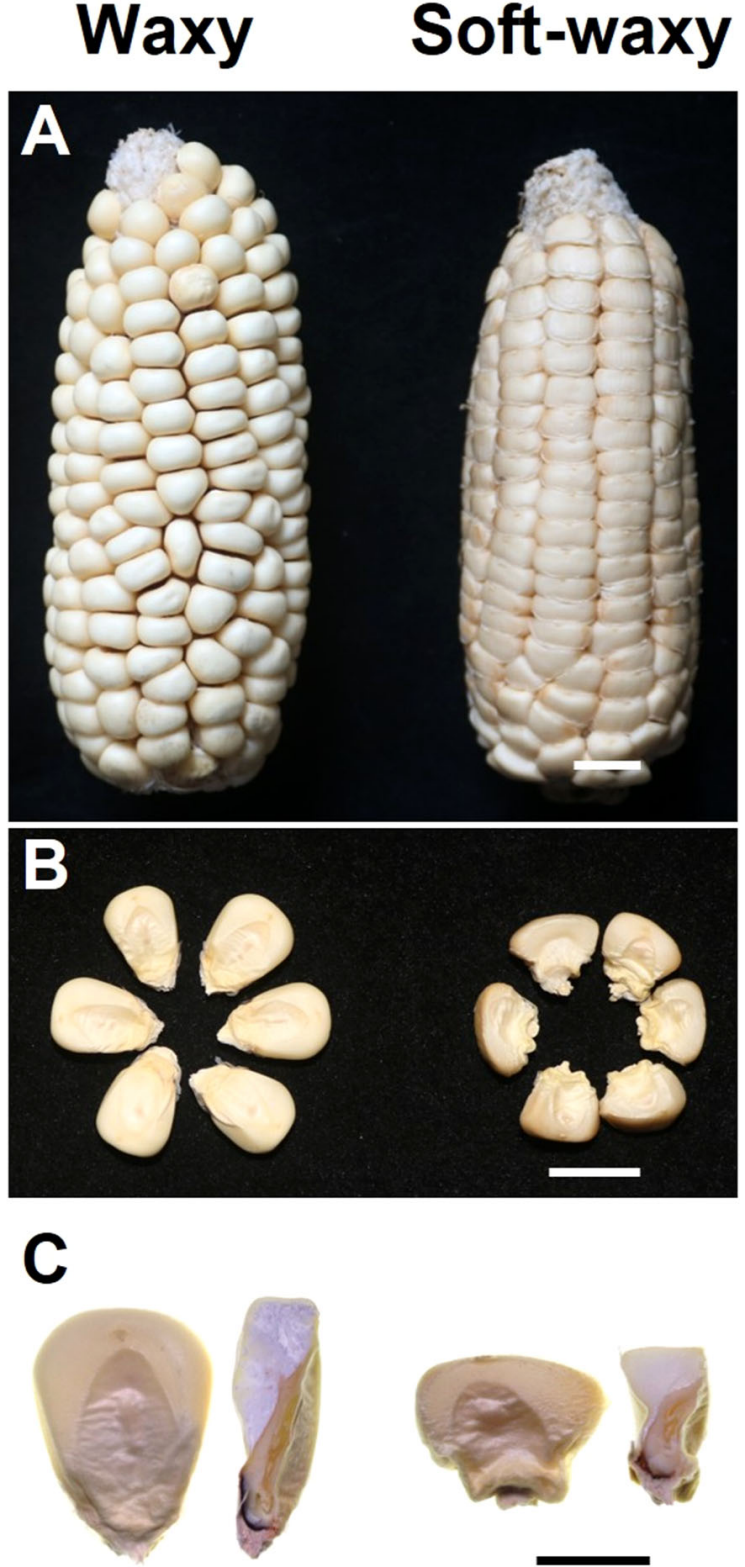
Phenotypic observation of waxy and soft-waxy maize kernels. **(A)** Mature ear, **(B)** Phenotypes of kernels and **(C)** Cross section of waxy and soft-waxy kernels. Bar = 0.5 cm.

Based on these evaluations, inbred line No. 5, characterized by low hardness and high stickiness, was designated “soft-waxy maize”. In contrast, inbred line No.1, which exhibited the highest hardness, served as the control for subsequent analyses and was designated “waxy maize”. The soft-waxy maize had a smaller kernel, wrinkled kernel surface and more opaque endosperm phenotype while the waxy maize showed a plump and smooth kernel phenotype (**Figures 1A–C**). All plants were grown in the Southwest University Experimental Farm (Chongqing, China) from March to August in2023, and2024. Kernels were stripped off the ears and processed for starch isolation, as well as for structural and functional analyses at maturity.

### 2.2 Starch isolation

Maize kernels were harvested upon maturity (50 DAP). For starch isolation, 100 g dried kernels with embryo and pericarp removed were steeped in 500 mL of a 1 g/L NaHSO_3_ solution for 48 h at room temperature. Starch was then isolated following a previously described method (Lu and Lu, 2012).

### 2.3 Proximate composition measurement

The starch content of maize kernels was quantified using the anthrone–sulfuric acid method (Hansen and Moller, 1975). Starch amylose content was determined following an established protocol (Garg and Jana, 2011). Protein and lipid contents in the starches were measured according to the approved American Association of Cereal Chemists (AACC) method (AACC, 2000). Swelling power of the starches was assessed based on previously described methods (Chen et al., 2018). Starches were heated at 95 °C, after which solubilized and undissolved fractions were separated and weighed to calculate the swelling power.

### 2.4 Pasting properties

The pasting properties of the two types of maize starch were determined by a rapid visco-analyzer (RVA-4500, Newport Scientific Pty. Ltd., Sidney, Australia) following a previously described method (Lu and Lu, 2012).

### 2.5 Thermal properties

The thermal characteristics of the two maize starches were evaluated using differential scanning calorimetry (Model 200 F3 Maia, NETZSCH, Germany) according to a reported method (Shi et al., 2018). The thermal transitions of starch were defined as the onset temperature (T_o_), peak gelatinization temperature (T_p_), conclusion temperature (T_c_), and gelatinization enthalpy (ΔH_gel_).

### 2.6 Scanning electron microscopy

The morphology of the two maize starches was examined using a high-resolution field-emission scanning electron microscope (Regulus8100, Hitachi, Tokyo, Japan). Starch samples were uniformly adhered to conductive double-sided sticky tape, sputter-coated with gold, and imaged under the scanning electron microscope at room temperature. Observations were conducted at magnifications of2000, × and1000, × under an accelerating voltage of 3.0 kV.

### 2.7 Molecular weight and amylopectin chain length distributions

Starch samples (10 mg) were resuspended in 5 mL of pure water and heated in a boiling water bath for 60 min. Subsequently, the solution was treated with 10 μL isoamylase (1400 U; Sigma, St. Louis, MO, USA), 10 μL sodium azide (2% w/v; Sangon Biotech, China), and 50 μL acetate buffer (0.6 M, pH 4.4; Sigma) to enzymatically debranch the starch.

The molecular weight distribution of two starch samples was determined by gel permeation chromatography on a PL-GPC 220 system (Agilent Technologies UK Limited, Shropshire, UK) equipped with three tandem columns (300 × 8 mm; Shodex OH-pak SB-805, 804, and 803; Showa Denko K.K., Tokyo, Japan) following previously described methods (Cai et al., 2014; Lin et al., 2016a). All experiments were performed in duplicate.

Amylopectin chain length distribution was analyzed by high-performance anion-exchange chromatography on a Thermo ICS5000 ion-chromatography system (ICS500+, Thermo Fisher Scientific, Waltham, MA, USA) according to the procedure described by Liu et al. (2007).

### 2.8 Transcriptomic analysis

Endosperm samples were collected from waxy and soft-waxy maize at 15 and 25 DAP. with three biological replicates per time point and genotype. Total RNA extraction and RNA-sequencing were conducted by Beijing BioMarker Technologies Corporation (Beijing, China) based on the Illumina sequencing platform. After quality control, a total of 86.40 Gb of clean reads were retained following the removal of low-quality and contaminated raw reads. Clean reads were aligned to reference genome (Zm_B73_REFERENCE_NAM_5.0 from maizeGDB) via HISAT2 system. Differentially expressed genes (DEGs) between two groups were identified with DESeq2, applying thresholds of log2 FC ≥ 1, *P*-value < 0.01 to control the false discovery rate (FDR). Two complementary comparisons were conducted: genotype-dependent differences (soft-waxy *vs*. waxy at each time point) and developmental regulation (15 *vs*. 25 DAP within each genotype).

### 2.9 Metabolic analysis

Endosperm samples were collected from waxy and soft-waxy maize at 15 and 25 DAP for metabolite profiling via liquid chromatography–mass spectrometry (LC-MS). Three biological replicates analyzed for each sample group. Sample preparation, extraction, metabolite identification, and quantification were conducted at Beijing BioMarker Technologies Corporation (Beijing, China) according to their standard procedures. The LC-MS system comprised an Acquity I-Class PLUS ultra-high-performance liquid chromatograph (UPLC) coupled to a Xevo G2-XS QTof high-resolution mass spectrometer (WATERS, USA). Following metabolite identification, orthogonal partial least squares-discriminant analysis (OPLS-DA) was conducted. Metabolites satisfying the criteria of log2 FC ≥ 1, *P*-value < 0.05, and variable importance in projection (VIP) score ≥ 1 were considered differentially accumulated metabolites (DAMs). Pathway enrichment analysis of the DAMs were performed based on the Kyoto Encyclopedia of Gene and Genomes (KEGG) database using the hypergeometric distribution test.

### 2.10 Assay of GBSS and SSS activity

Endosperm samples (100 mg) were collected from waxy and soft-waxy maize at 15 and 25 DAP ground in liquid nitrogen were extracted with 100 μL of ice-cold extraction buffer containing 100 mM HEPES-NaOH (pH 7.4), 8 mM MgCl_2_, 50 mM 2-mercaptoethanol, 2 mM EDTA, 12.5% (v/v) glycerol, and 5% (w/v) insoluble PVPP. After incubation on ice for 5 min, the homogenate was centrifuged at 13,000 g for 15 min at 4 °C. The pellet was resuspended in extraction buffer for GBSS assay, while the supernatant was re-centrifuged and used for SSS assay. GBSS and SSS activities were assayed according to Nakamura et al. (1989) with modifications. The reaction mixture (20 μL enzyme extract + 36 μL Mixture A) contained 50 mM HEPES-NaOH (pH 7.5), 1.6 mM ADPG, 0.7 mg amylopectin, and 15 mM DTT. After incubation at 30 °C for 20 min and boiling for 1 min, 20 μL Mixture B (50 mM HEPES-NaOH pH 7.5, 4 mM PEP, 200 mM KCl, 10 mM MgCl_2_, 1.2 U pyruvate kinase) was added and incubated at 30 °C for 20 min. Following boiling and centrifugation, 60 μL supernatant was mixed with 43 μL Mixture C (50 mM HEPES-NaOH pH 7.5, 10 mM glucose, 20 mM MgCl_2_, 2 mM NADP, 1.4 U hexokinase, 0.35 U G6PDH). After 10 min at 30 °C, absorbance was measured at 340 nm. Boiled enzyme extract served as control.

### 2.11 Statistical analysis

Data presented in figures and tables represent the means of three replicate measurements/experiments. Analysis of variance was performed using LSD test with the data processing system (version 7.05); statistical significance was judged at the *P* < 0.05 level.

## 3 Results

### 3.1 Physiochemical and pasting properties

The physiochemical characteristics of waxy and soft-waxy maize starches were presented in **Table 2**. The amylose content was only 3.2% and 2.1% in waxy and soft-waxy maize starch, respectively, classifying these lines as low-amylose-content maize types. The protein content was similar in the two maize starches. The soft-waxy maize starch had lower swelling power when compared with those of the waxy maize starch, suggesting inherent differences in granular structure and hydration behavior.

**Table 2:** Physicochemical properties of waxy and soft-waxy maize starches.

Sample	Starch (%)	Amylose content (%)	Protein (%)	Swelling power (g/g)
W	98.6 ± 0.2^a^	3.2 ± 0.2^a^	0.32 ± 0.1^a^	47.8 ± 1.5^a^
SW	97.5 ± 0.2^b^	2.1 ± 0.2^b^	0.33 ± 0.1^a^	46.6 ± 0.6^b^

Values mean ± SD indicate the replicates of three experiments. Values in the same columns with different superscript letters are significantly different (*P* < 0.05). W, waxy maize starch; SW, soft-waxy maize starch.

**Figure 2:**
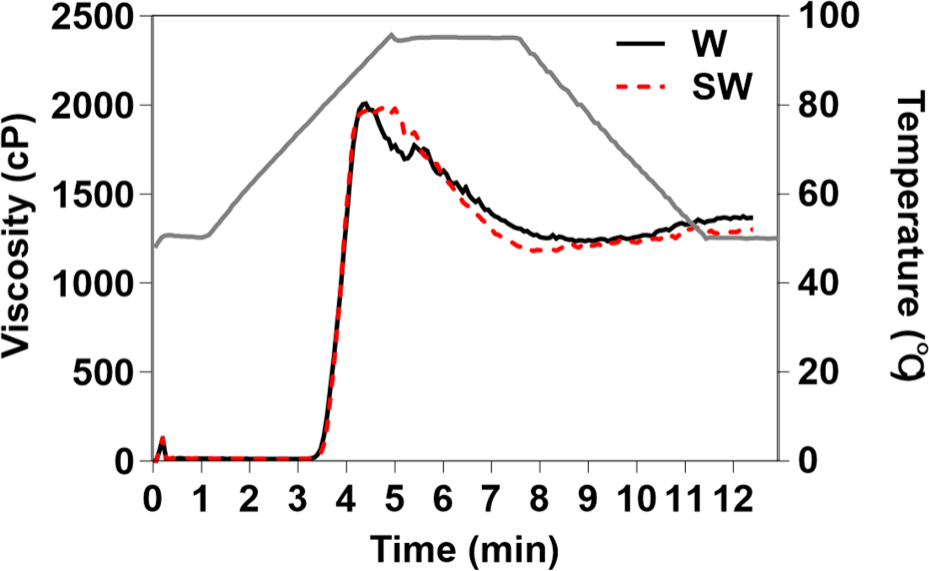
Pasting profiles of waxy and soft-waxy maize starches. W, waxy maize starch; SW, soft-waxy maize starch.

The pasting profiles varied between the two maize starches (**Table 3**, **Figure 2**). While no difference was observed in pasting temperature, the peak viscosity time was longer for soft-waxy maize starch (4.8 min) than for waxy maize starch (4.3 min). A shorter peak viscosity time generally reflects better swelling property and a faster pasting process. Therefore, the soft-waxy maize starch exhibited comparatively weak expansion performance during pasting. All recorded viscosity parameters, including peak, trough, breakdown, final, and setback viscosities, were lower in soft-waxy maize starch than in waxy maize starch (**Table 3**). The pasting properties, particularly the low breakdown and setback viscosities of soft-waxy maize starch, may contribute to its stable viscoelasticity and eating quality, associated with a softer gel texture and delayed hardening in both hot and cooled states. The results suggested that pasting properties, particularly breakdown and setback viscosity, may serve as useful reference indicators for evaluating the edible and processing characteristics of maize starch.

**Table 3:** Pasting properties of waxy and soft-waxy maize starches.

Sample	PT (°C)	Peak time (min)	Viscosity (cP)				
			PV	TV	BV	FV	SV
W	78.2 ± 0.4^a^	4.3 ± 0.1^b^	2130 ± 24^a^	1248 ± 13^a^	882 ± 21^a^	1388 ± 21^a^	140 ± 8^a^
SW	78.6 ± 0.1^a^	4.8 ± 0.2^a^	1969 ± 16^b^	1200 ± 20^b^	769 ± 36^b^	1314 ± 13^b^	114 ± 7^b^

Values mean ± SD indicate the replicates of three experiments. Values in the same columns with different superscript letters are significantly different (*P* < 0.05). W, waxy maize starch; SW, soft-waxy maize starch. PT, peak temperature; PV, peak viscosity; TV, trough viscosity; BV, breakdown viscosity; FV, final viscosity; SV, setback viscosity.

### 3.2 Thermal properties

The gelatinization temperatures, including the onset (T_o_), peak (T_p_), and conclusion temperature (T_c_), reflect the energy required to initial and complete the gelatinization process. In this study, the soft-waxy maize starch exhibited significantly higher T_o_, T_p_, and T_c_ values compared to waxy maize starch, indicating greater thermal stability of its crystalline regions. While the gelatinization enthalpy (ΔH_gel_), which corresponds to the overall energy needed for crystal melting, showed no significant difference between the two maize starches (**Table 4**). The results suggested that the distinct thermal properties of soft-waxy maize starch are likely attributable to specific characteristics of its molecular structure rather than overall degree of crystalline.

**Table 4:** Thermal properties of waxy and soft-waxy maize starches.

Sample	T_o_ (°C)	T_p_ (°C)	T_c_ (°C)	ΔH_gel_ (J/g)
W	71.9 ± 0.1^b^	77.0 ± 0.0^b^	84.0 ± 0.2^b^	15.5 ± 0.1^a^
SW	73.2 ± 0.1^a^	78.3 ± 0.1^a^	85.1 ± 0.1^a^	15.7 ± 0.1^a^

Values mean ± SD indicate the replicates of three experiments. Values in the same columns with different superscript letters are significantly different (*P* < 0.05). W, waxy maize starch; SW, soft-waxy maize starch; T_o_, onset temperature; T_p_, peak temperature; T_c_, conclusion temperature; ΔH_gel_, enthalpy of gelatinization (based on starch weight).

### 3.3 Morphological and molecular weight distribution analysis

**Figure 3:**
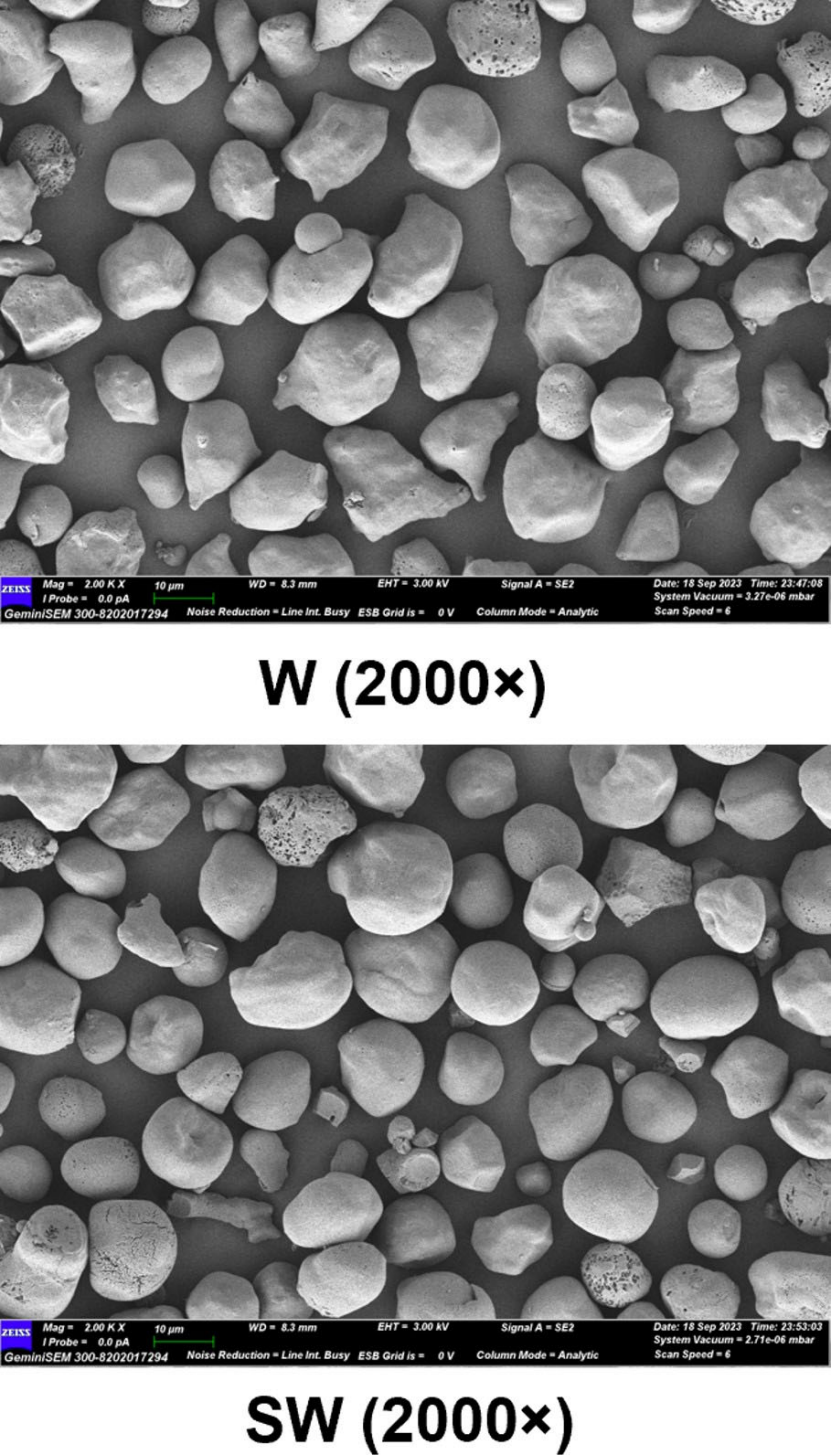
Scanning electron microscope images of granule morphology of waxy and soft-waxy maize starches. W, waxy maize starch; SW, soft-waxy maize starch.

Scanning electron microscopy (SEM) was performed to examine starch granule morphology. Both waxy and soft-waxy maize starches exhibited smooth surfaces with polygonal or irregular shapes **(Figure 3**), indicating successful starch isolation. No obvious morphological differences were observed between the two genotypes at the granule level.

The soft-waxy maize starch displayed higher weight-average molecular weight (Mw) and number-average molecular weight (Mn) compared to waxy maize starch, alongside a lower polydispersity index (Mw/Mn) (**Table 5**). Given that a higher amylopectin content generally elevates Mn and Mw, and that the polydispersity index reflects the Mw distribution width of a polymer, as the width of the distribution is positively correlated with the Mw/Mn ratio (Han and Lim, 2004), these results indicate that the soft-waxy maize starch possesses not only larger average molecular size but also a more uniform molecular weight distribution. This refined molecular structure may be associated with its unique physicochemical and superior palatability properties.

**Table 5:** Molecular weight parameters of waxy and soft-waxy maize starches.

Sample	Mw (10^7^ g/mol)	Mn (10^7^ g/mol)	Polydispersity (Mw/Mn)
W	2.097 ± 0.16^b^	1.570 ± 0.07^b^	1.336 ± 0.01^a^
SW	2.397 ± 0.08^a^	2.028 ± 0.21^a^	1.187 ± 0.09^b^

Mw, average molecular weight; Mn, number average molecular weight. Values mean ± SD indicate the replicates of three experiments. Values in the same columns with different superscript letters are significantly different (*P* < 0.05). W, waxy maize starch; SW, soft-waxy maize starch; Mw, weight-average molecular weight; Mn, number-average molecular weight.

### 3.4 Chain-length distribution analysis

**Figure 4:**
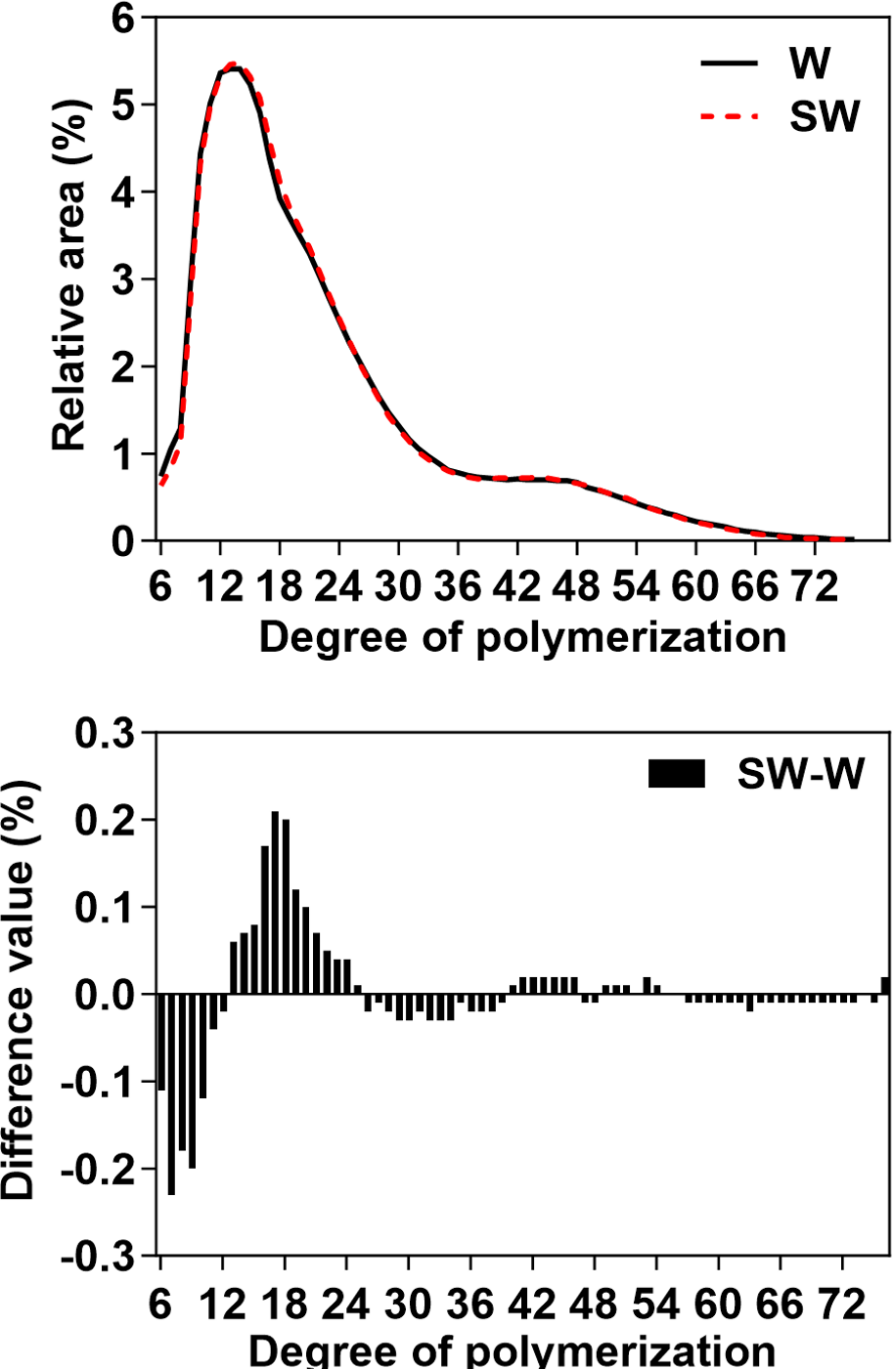
Amylopectin chain length distribution of waxy and soft-waxy maize starches. The curves on the left indicate the distribution of amylopectin chains with different degrees of polymerization (DP). The bars on the right represent the difference in values of soft-waxy and waxy maize starches. W, waxy maize starch; SW, soft-waxy maize starch.

The side chains of amylopectin are classified on the basis of the degree of polymerization DP into A (DP 6–12), B1 (DP 13–24), B2 (DP 25–36), and B3 (DP > 36) chains (Hanashiro et al., 1996). The chain-length distributions of the two maize starches are presented in **Figure 4**. Compared with the waxy maize starch, the soft-waxy maize starch showed an increased proportion of B1 chains, along with decreased proportions of A and B2 chains. B1 chains constituted the most abundant category in both maize starches, indicating its potential dominant role in determining starch properties, relative to the less abundant B2 and B3 chains. The increase in B1 chains and reduction in A chains may account for the higher gelatinization temperature observed in soft-waxy maize starch.

### 3.5 Transcriptomic analysis and expression of genes in the maize starch biosynthesis pathway

**Figure 5:**
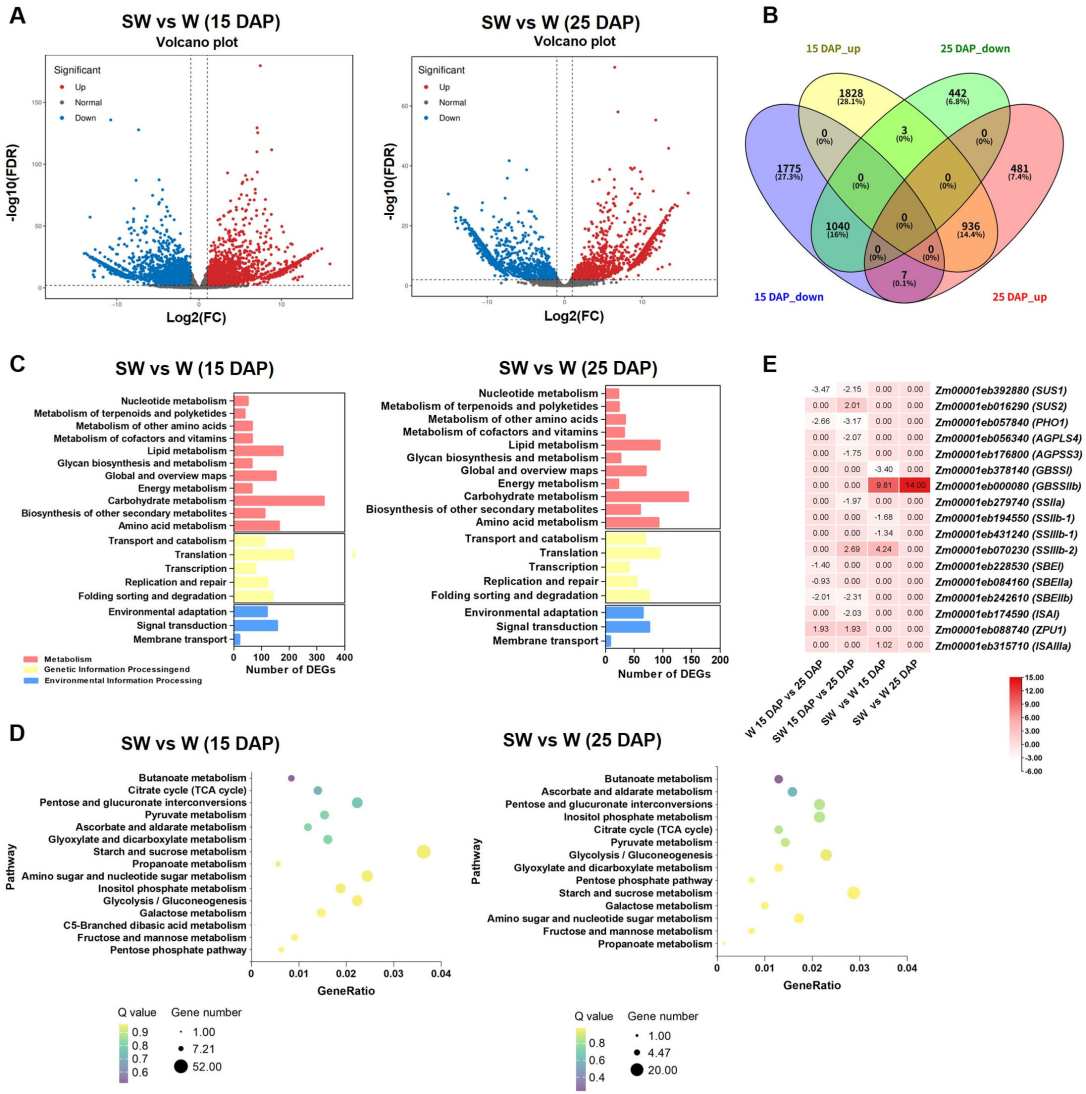
Differentially expressed genes (DEGs) between waxy and soft-waxy maize kernels at each developmental stage and Kyoto Encyclopedia of Genes and Genomes (KEGG) pathway analysis. **(A)** Volcano plots of DEGs. **(B)** Venn diagram showing the number of DEGs. **(C)** KEGG classification analysis. **(D)** KEGG enrichment analysis of the carbohydrate metabolism pathway. **(E)** Heat map of DEGs involved in starch biosynthesis. W, waxy maize; SW, soft-waxy maize.

To explore the molecular mechanisms underlying the structural differences in soft-waxy maize starch, we conducted transcriptome analysis of endosperm tissues from both waxy and soft-waxy maize at 15 and 25 DAP. Our analysis focused on two complementary comparisons: genotype-dependent differences (soft-waxy *vs*. waxy at each time point) and developmental regulation (15 *vs*. 25 DAP within each genotype). Direct comparison between genotypes revealed substantial transcriptional differences. At 15 DAP, a total of 5,589 differentially expressed genes (DEGs) between soft-waxy and waxy maize, comprising 2,767 up- and 2,822 downregulated genes. At 25 DAP, 2,909 DEGs were identified across the same developmental stages, with 1,424 up- and 1,485 downregulated genes (**Supplementary Table 1**, **Figures 5A, B**). KEGG pathway classification was performed to associate the DEGs with specific downstream processes to further explore the molecular basis of starch-related metabolic shifts. Carbohydrate metabolism was the most significantly enriched metabolism category at both developmental stages (**Figure 5C**). Within this category 324 DEGs were identified at 15 DAP, including 52 DEGs specifically enriched in starch and sucrose metabolism pathways. At 25 DAP, 144 DEGs were enriched in carbohydrate metabolism, including 20 DEGs enriched in starch and sucrose metabolism pathways (**Figures 5C, D**). These results suggest distinct structural and functional properties of soft-waxy and waxy maize starches are associated with genotype-specific transcriptional reprogramming of carbohydrate metabolism, particularly during early endosperm development.

Further screening identified DEGs related to starch biosynthesis enzymes at each developmental stage by comparing soft-waxy and waxy maize at each time points (SW *vs*. W at 15 DAP and SW *vs*. W at 25 DAP) (**Figure 5E**; **Supplementary Table 2**). Three SSS genes, *SSIIIb-2* (4.24, 2.68E-05) was upregulated in soft-waxy maize at 15 DAP. *SSIIIb-1* (1.34, 8.17E-03) and *SSIIb-1* (1.68, 9.59E-03) were downregulated in soft-waxy maize at 15 DAP. No differences were detected at 25 DAP for either gene. One DBE gene, *ISAIIIa* (1.02, 1.32E-04) showed modest upregulation in soft-waxy maize at 15 DAP, with no difference at 25 DAP. Two GBSS genes, *GBSSI* (-3.40, 3.27E-15) was significantly downregulated in soft-waxy maize at 15 DAP, with no difference at 25 DAP; *GBSSIIb* showed strong upregulation in soft-waxy maize at 15 DAP (9.81, 1.25E-15) and 25 DAP (14.00, 9.13E-27). Notably, none of the *GBSS* genes exhibited developmental regulation in either genotype.

Developmental comparisons within each genotype revealed additional regulatory differences (**Supplementary Table 1**; **Supplementary Figure 1**). 6 and 10 DEGs related to starch biosynthesis enzymes were identified in waxy and soft-waxy maize during endosperm maturation in waxy maize (W 15 DAP *vs*. 25 DAP) and soft-waxy maize (SW 15 DAP *vs*. 25 DAP), respectively (**Figure 5E**; **Supplementary Table 2**). In the soft-waxy maize, two AGPase genes, *AGPLS4* (log_2_FC, FDR -2.07, 1.32E-08) and *AGPSS3* (*Bt2*; -1.75, 1.05E-03), were downregulated. Among SSS genes, *SSIIa* (*Su2*; -1.97, 1.50E-06) was downregulated and *SSIIIb-2* (2.69, 8.53E-06) was upregulated. Three SBE genes, *SBEI* (-1.40, 6.44E-03), *SBEIIa* (-0.93, 4.24E-03) and *SBEIIb* (*Ae1*; -2.01, 6.48E-03), were downregulated in waxy maize. In comparison, only *SBEIIb* was downregulated in soft-waxy maize, and its downregulation was greater (-2.31, 7.61E-09). One DBE gene, *ZPU1* was upregulated in both waxy (1.93, 5.14E-05) and soft-waxy (1.93,1.22E-03) maize. Another DBE gene, *ISAI* (*Su1*; -2.03, 1.07E-08), was downregulated in soft-waxy maize.

Collectively, the transcriptome analysis revealed distinct regulatory patterns underlying starch biosynthesis in waxy and soft-waxy maize. The DEGs identified, particularly those involved in amylopectin synthesis, are implicated in the modulation of starch composition and functional properties. Further investigation is required to precisely elucidate how these genes affect the maize starch properties, as well as to quantify correlations between gene expression levels and compositional proportions or contents.

### 3.6 GBSS and SSS enzyme activities

**Figure 6:**
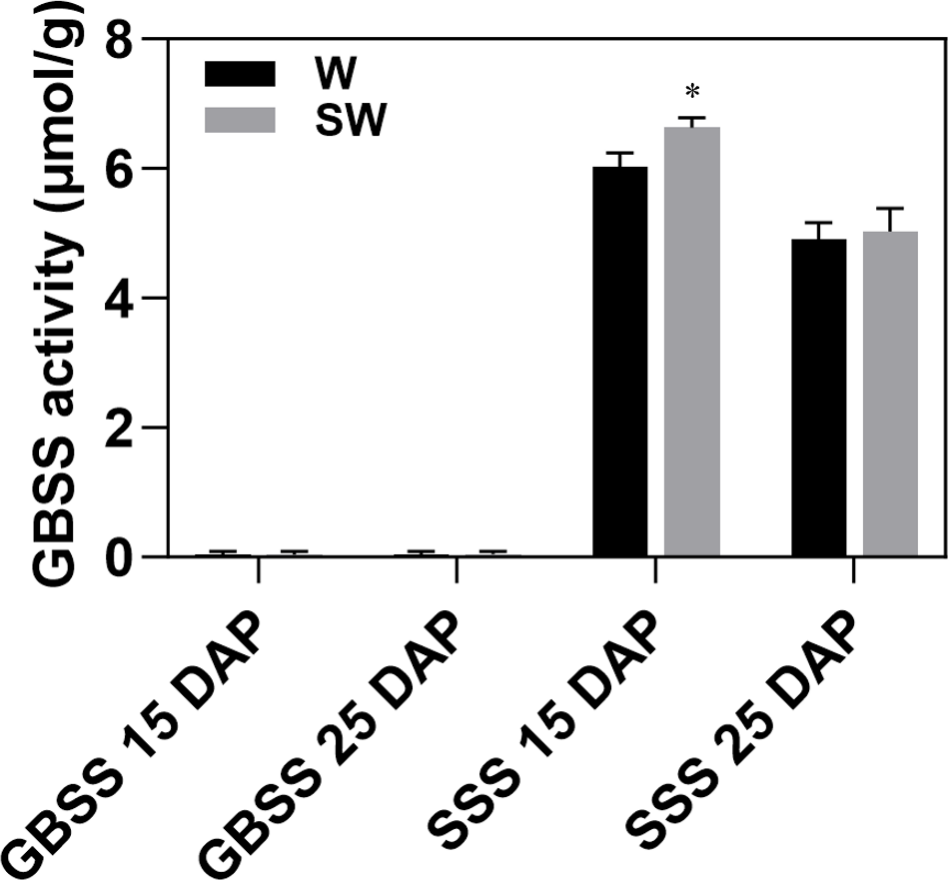
GBSS and SSS activities in developing endosperms of waxy and soft-waxy maize at 15 and 25 DAP. Values are means ± SD (n = 3). Different letters indicate significant differences at *P < 0.05. W, waxy maize; SW, soft-waxy maize.

GBSS and SSS activities were assayed in waxy and soft-waxy maize endosperms at 15 and 25 DAP. GBSS activity was nearly undetected in both genotypes at both stages, with no significant differences between genotypes or stages (**Figure 6**). SSS activity showed a genotype-specific difference at 15 DAP, with significantly higher activity in soft-waxy than in waxy. However, no difference was observed at 25 DAP (**Figure 6**). These results indicate that enhanced SSS activity in soft-waxy occurs specifically during early endosperm development, which may contribute to the altered amylopectin structure in soft-waxy starch.

### 3.7 Combined transcriptome and metabolome analysis

**Figure 7:**
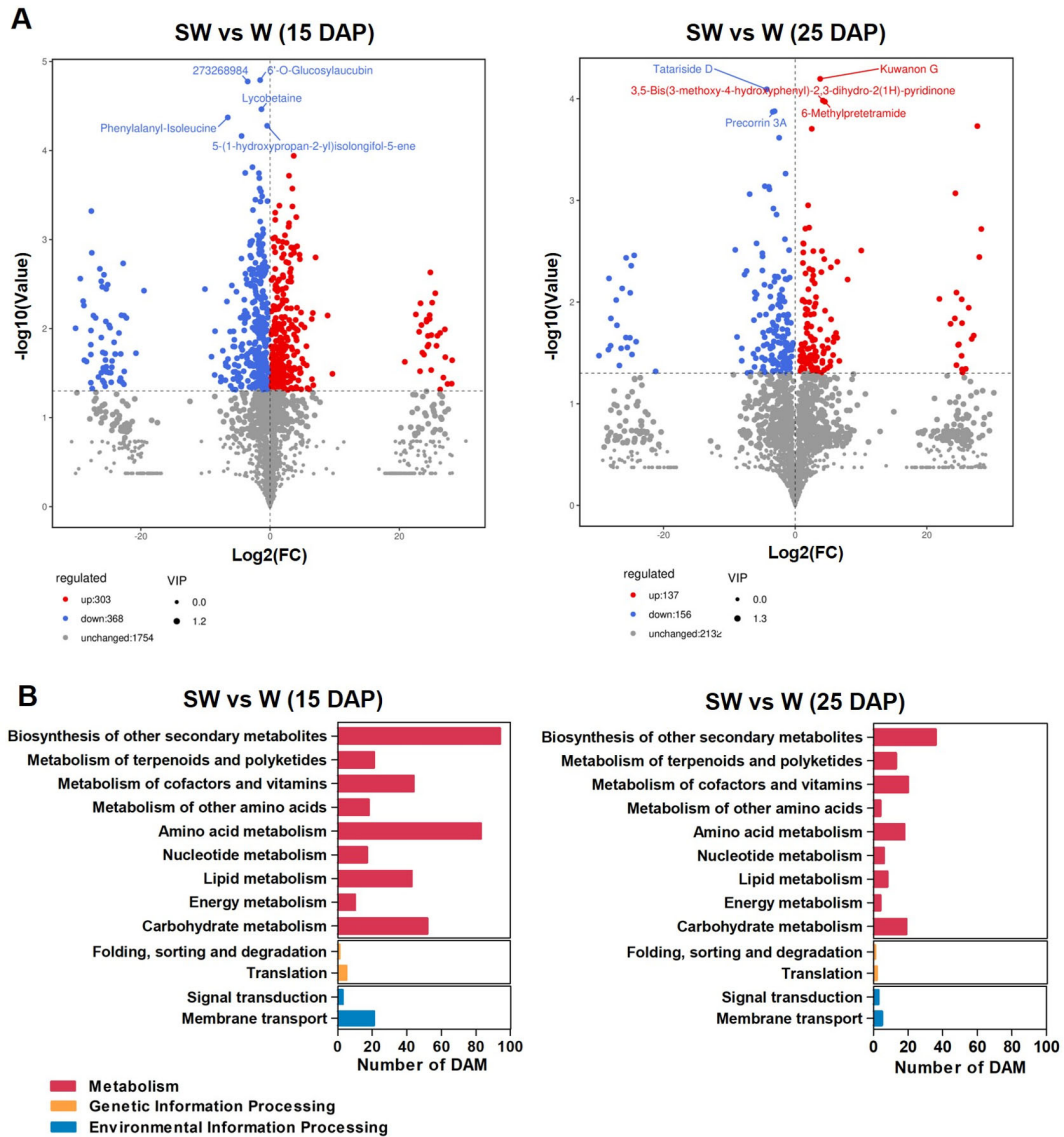
Differentially accumulated metabolites (DAMs) between waxy and soft-waxy maize kernels at each developmental stage and Kyoto Encyclopedia of Genes and Genomes (KEGG) pathway analysis. **(A)** Volcano plot of DAMs. Red and blue dots represent up- and downregulated metabolites, respectively. **(B)** KEGG classification analysis. W, waxy maize; SW, soft-waxy maize.

Certain metabolites present in the endosperm of waxy maize may serve as key factors influencing texture and flavor. Using LC–MS-based metabolite profiling, we quantitatively analyzed over 2,400 metabolites from waxy and soft-waxy maize endosperms at 15 and 25DAP. At 15 DAP, 671 DAMs (303 up, 368 down) were identified between soft-waxy and waxy maize, and 293 DAMs (137 up, 156 down) at 25 DAP (**Figure 7A**; **Supplementary Table 3**). KEGG pathway classification revealed significant enrichment of DAMs in multiple metabolic categories. Specifically, at 15 and 25 DAP, respectively, 95 and 36 DAMs enriched in secondary metabolites biosynthesis pathway (e.g., isoquinoline alkaloid biosynthesis, phenylpropanoid biosynthesis); 72 and 18 DAMs enriched in amino acid metabolism (e.g., tryptophan metabolism, cysteine and methionine metabolism); 37 and 20 DAMs enriched in metabolism of cofactors and vitamins (e.g., nicotinate and nicotinamide metabolism); and 38 and 19 DAMs enriched in carbohydrate metabolism (e.g., amino sugar and nucleotide sugar metabolism, starch and sucrose metabolism) (**Figure 7B**; **Supplementary Table 4**).

To better understand the relationship between the metabolites and genes underlying starch biosynthesis differences in the two waxy maize endosperms, we performed an integrative transcriptome and metabolome analysis. At 15 DAP, jointly enriched pathways included starch and sucrose metabolism (52 DEGs, 4 DAMs), amino sugar and nucleotide sugar metabolism (35 DEGs, 10 DAMs), glycolysis/gluconeogenesis (32 DEGs, 2 DAMs), phenylpropanoid biosynthesis (57 DEGs, 5 DEMs), and glutathione metabolism (35 DEGs, 2 DAMs). At 25 DAP, similar pathways were enriched, including starch and sucrose metabolism (20 DEGs, 1 DAM), glycolysis/gluconeogenesis (16 DEGs, 2 DAMs), and phenylpropanoid biosynthesis (24 DEGs, 2 DAMs) (**Supplementary Table 5**). Metabolites in these pathways were detailed in **Supplementary Table 6**. Among them, D-glucose (log_2_FC = 0.79) and D-fructose 6-phosphate (0.87) were elevated in soft-waxy maize at 15 DAP, while isomaltose (1.81) accumulated at 25 DAP. In starch and sucrose metabolism pathway, *SUS1* (*Sucrose Synthase 1*) were downregulated in both waxy (-3.47, 8.64E-10) and soft-waxy (-2.15, 9.82E-07) maize, while *SUS2* (2.01, 3.53E-06) was upregulated in soft-waxy maize (**Figure 5D**; **Supplementary Table 2**). Beyond carbohydrate metabolism, metabolites within the phenylpropanoid pathway were also identified. 4-coumaryl alcohol (23.07) and ferulate (1.71) accumulated preferentially in soft-waxy maize at 15 DAP, while 1-O-sinapoyl-beta-D-glucose (6.20) was elevated at 25 DAP. Taken together, these results underscore that the identified DEGs and associated metabolites, spanning starch/sugar metabolism, amino acid pathways, and secondary compound synthesis, are correlated with the unique sensory attributes, suggesting their potential associations with kernel quality, including their taste, texture, flavor, and overall palatability.

## 4 Discussion

### 4.1 Linking starch structure to unique functional properties

The distinct functional properties of soft-waxy maize starch can be interpreted in light of its multi-scale structural features. The lower swelling power of soft-waxy maize starch (**Table 2**) is consistent with restricted granule expansion during heating, which is influenced by the interplay between crystalline and amorphous regions within the granule (Tester and Morrison, 1990; Heeres et al., 1998). This restricted swelling may be related to its unique molecular structure: the higher weight-average molecular weight (Mw) and number-average molecular weight (Mn), alongside a lower polydispersity index (Mw/Mn) (**Table 5**), suggest a more uniform molecular weight distribution. This uniformity could promote stronger internal chain interactions, which potentially limits water ingress and granule swelling (Han and Lim, 2004). However, direct evidence linking these molecular features to swelling behavior would require further investigation, such as controlled fractionation or reconstitution experiments.

This molecular architecture is associated with the observed reduction in all pasting viscosity parameters, including peak, trough, and final viscosities (**Table 3**). The pasting profile provides insights into the starch’s functional behavior. The breakdown value, calculated as the difference between peak and trough viscosity, is commonly used as an indicator of hot paste stability (Chen et al., 2015). A lower breakdown value in soft-waxy maize starch suggests that the starch granules disintegrated gradually after peak viscosity was achieved, resulting in a slower decline in paste viscosity and potentially enhanced stability of swollen granules under shear and heat, a trait generally considered desirable for food processing. Most importantly, the significantly lower setback value indicates a reduced tendency for starch molecule reassociation upon cooling, which is often associated with its softer gel texture and delayed hardening after cooling. This finding is consistent with reports in soft rice, where similarly low setback viscosity has been linked to favorable eating quality that remains stable at both hot and cold serving temperatures (Wang et al., 2020). Therefore, pasting properties, particularly breakdown and setback viscosity, may serve as informative indicators of starch functional behavior, though their predictive value for eating quality requires further investigation incorporating sensory analysis.

The altered amylopectin fine structure provides a possible explanation for the observed thermal properties. The reduction in short A chains (DP 6–12) and the increase in B1 chains (DP 13–24) in soft-waxy maize starch (**Figure 4)** are consistent with its higher gelatinization temperatures (**Table 4**). Previous studies have shown that the crystalline lamellae formed by longer B1 chains are more ordered and stable, requiring more energy (higher temperature) to initiate melting compared to structures rich in short A chains (Lin et al., 2016b). While this structural difference offers a compelling hypothesis for the thermal behavior of soft-waxy starch, establishing a causal relationship will require further validation.

### 4.2 Potential molecular mechanisms of starch biosynthesis and regulation

The transcriptome analysis reveals the coordinated genetic changes associated with the unique starch phenotype in soft-waxy maize. Among soluble starch synthase genes, *SSIIIb-2* emerged as a key candidate gene. Cross-genotype comparison revealed that *SSIIIb-2* was significantly up-regulated in soft-waxy maize at 15 DAP, with no difference detected at 25 DAP. Developmental analysis further showed that *SSIIIb-2* was significantly upregulated from 15 DAP to 25 DAP exclusively in soft-waxy maize, while remaining unchanged in waxy maize throughout development (**Figure 5E**). This dual pattern, genotype difference at an early developmental window and soft-waxy-specific developmental regulation, establishes *SSIIIb-2* as a prime candidate influencing starch structure. *SSIIIb-2* encodes a starch synthase III isoform responsible for elongating B1 and B2 chains of amylopectin (Jeon et al., 2010; Lin et al., 2011; Irshad et al., 2021), consistent with the increased proportion of medium-length B1 chains observed in soft-waxy starch (**Figure 4**). Conversely, *SSIIIb-1* and *SSIIb-1* were down-regulated in soft-waxy maize specifically at 15 DAP, and *SSIIa*, which preferentially extends shorter A chains (Irshad et al., 2021), was significantly downregulated from 15 DAP to 25 DAP only in soft-waxy maize (**Figure 5E**). This expression pattern, *SSIIa* downregulation coupled with *SSIIIb-2* upregulation, is consistent with a shift favoring the accumulation of medium-length B1 chains at the expense of short A chains, matching the observed chain-length alterations in soft-waxy starch. SSS activity showed a genotype-specific temporal pattern: significantly higher in soft-waxy than waxy maize at 15 DAP, but no difference at 25 DAP (**Figure 6**). This early-stage enhancement aligns with *SSIIIb-2* upregulation and likely contributes to the altered amylopectin structure in soft-waxy starch, including increased B1 chains and reduced A chains.

In maize endosperm, SBEIIb isoform plays a dominant role, and its activity is positively correlated with the proportion of chains with DP < 12 of amylopectin and negatively correlated with T_o_ (Tanaka et al., 2004). In this study, *SBEIIb* was more strongly downregulated in soft-waxy maize during development (**Figure 5E**), explaining its reduced A chain proportion and higher T_o_. *SBEI* showed downregulation exclusively in waxy maize potentially accounting for its lower proportion of B1 chains relative to soft-waxy maize. Among debranching enzyme genes, *ISAIIIa* showed genotype-specific up-regulation in soft-waxy maize at 15 DAP, while *ISA1* (*su1*) exhibited soft-waxy-specific downregulation at 25 DAP (**Figure 5E**). These changes may refine amylopectin branched structure and influence crystallinity (Qu et al., 2018). Additionally, downregulation of key AGPase subunit genes (*AGPLS4* and *Bt2*) only in soft-waxy maize during mid-grain filling (**Figure 5E**) suggests a possible moderated flux of ADP-glucose, the primary substrate for starch synthesis (Liu et al., 2023), potentially influencing the availability of primers for amylopectin assembly.

Notably, GBSS activity was nearly undetectable in both genotypes at 15 and 25 DAP, with no significant differences between lines or stages (**Figure 6**), confirming the waxy phenotype. Although *GBSSI* and *GBSSIIb* transcripts were detectable (**Figure 5E**), the absence of enzyme activity indicates that the *wx* mutation affects protein function rather than transcription, this result consistent with previous report (Ding et al., 2009).

Beyond the core starch biosynthesis genes discussed above, the transcriptomic analysis revealed widespread transcriptional regulation during endosperm development. Thousands of DEGs were identified in both genotypes (**Figure 5A**), with KEGG enrichment analysis highlighting significant changes in multiple functional categories, including carbohydrate metabolism, amino acid metabolism, and secondary metabolite biosynthesis pathways (**Figures 5B, C**). These broader metabolic shifts may indirectly influence starch properties by altering carbon flux, energy status, or precursor availability.

### 4.3 Integrated omics revealed the metabolic candidate regulatory network

Integrated metabolomic analysis revealed shifts in key metabolic pathways might potentially contribute to the observed starch structural phenotype. Within the starch and sucrose metabolism pathway, *SUS1* was down-regulated in both genotypes, with greater magnitude in waxy (-3.47) than soft-waxy maize (-2.15). In contrast, *SUS2* showed up-regulation exclusively in soft-waxy maize at 25 DAP (2.01) (**Supplementary Table 2**). This differential regulation suggests genotype-specific strategies for carbon partitioning during grain filling (Bilska-Kos et al., 2020; Xiao et al., 2024). Consistent with this, D-glucose (0.79) and D-fructose 6-phosphate (0.87) were elevated in soft-waxy maize at 15 DAP, while isomaltose (1.81) accumulated at 25 DAP (**Supplementary Table 6**). Increased hexose phosphates may reflect enhanced carbon flux toward ADP-glucose production (Okita, 1992), potentially facilitating amylopectin synthesis with altered molecular weight properties (Han and Lim, 2004). Significant alterations were observed in phenylpropanoid biosynthesis. 4-Coumaryl alcohol (23.07) and ferulate (1.71) accumulated in soft-waxy maize at 15 DAP, while 1-O-sinapoyl-beta-D-glucose (6.20) was elevated at 25 DAP (**Supplementary Table 6**). These metabolites serve as lignin precursors, and their accumulation may modify endosperm cell wall properties, potentially influencing kernel texture (Fornalé et al., 2010). Reduced acetyl-CoA (-1.849) in soft-waxy maize may limit fatty acid synthesis and amylose-lipid complex formation, potentially influencing starch retrogradation properties (Sasaki et al., 2000).

Collectively, the metabolic reprogramming in maize endosperm that extends beyond primary carbohydrate metabolism into secondary metabolic pathways. While these observations generate intriguing hypotheses about potential contributors to kernel texture and flavor, sensory evaluation or biochemical characterization of relevant compounds are required for validation.

### 4.4 Potential implications and limitations

This study provides a comprehensive framework connecting genotype to starch structure and eating quality, offering a model that could potentially be applied in maize breeding. The key regulatory genes identified, particularly *SSIIIb-2*, could serve as potential candidate loci for developing molecular markers, enabling the precise selection of alleles for soft texture and palatability. Furthermore, the unique functional properties of soft-waxy maize starch, including low retrogradation and stable hot-paste viscosity, and delayed hardening, make it an ideal natural ingredient for food products. These traits are especially valuable in products requiring extended softness and moisture retention, such as baked goods, desserts, and ready-to-eat meals.

However, several limitations should be acknowledged. First, the detailed multi-omics comparisons were performed using only one soft-waxy and one waxy inbred line, it remains challenging to completely distinguish genotype-specific effects from those directly associated with the soft-waxy phenotype. The observed differences may partly reflect intrinsic genetic variation rather than universally linked mechanisms. Second, the candidate genes and pathways identified should therefore be interpreted as putative associations requiring further validation. Future studies should: (i) validate candidates in multiple independent genetic backgrounds; (ii) incorporate enzyme activity assays and proteomic analysis; and (iii) perform functional validation through gene editing or transgenic approaches.

## 5 Conclusions

**Figure 8:**
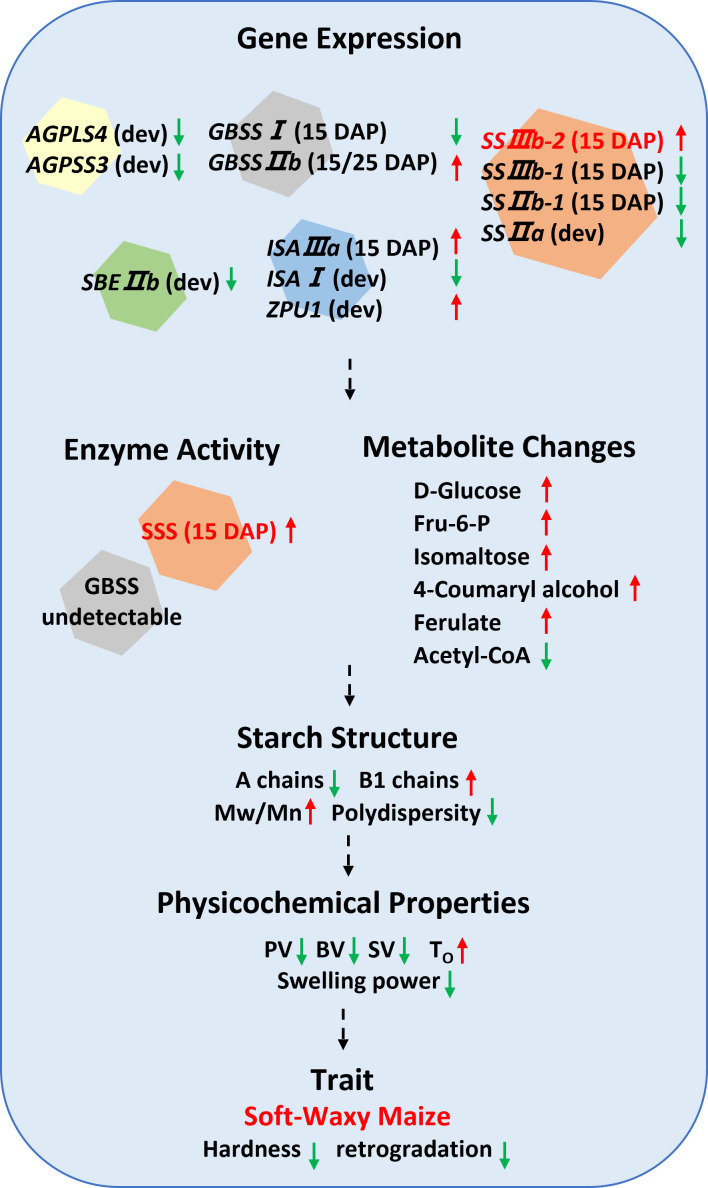
A proposed model integrating gene expression of starch biosynthesis pathways, metabolite changes, enzyme activity, and phenotypic outcomes in waxy and soft-waxy maize kernels. .

This study provides a multi-scale characterization of novel soft-waxy maize starch. Compared to waxy maize starch, the soft-waxy maize starch exhibited a lower amylose content, reduced swelling power, and distinctive pasting properties (e.g., lower peak, trough, final, and setback viscosities), and higher gelatinization temperatures. Starch molecular structure analysis revealed the higher Mw and Mn, lower polydispersity, and increased proportion of B1 chains in soft-waxy maize starch. Transcriptomic analyses identified differential expression of key starch biosynthetic genes, with *SSIIIb-2* as a candidate gene potentially contributing to the observed variations based on its genotype-specific and developmental expression pattern. The alterations in metabolite abundance within specific metabolic pathways may relate to kernel texture and flavor. A simplified model synthesizing these findings is proposed (**Figure 8).** However, this study has several limitations. First, the single-genotype comparison means our findings may not be broadly generalizable. Second, the omics data are correlative and should not be interpreted as causal. Third, the suggested connections between starch structure and eating quality require validation through sensory analysis. In summary, this work defines a unique waxy maize starch and provides a theoretical foundation for developing comprehensive evaluation methods, informing future efforts to dissect the molecular basis of eating quality in maize.

## Data Availability

The datasets generated and analyzed during the current study are available in the National Center for Biotechnology Information repository: https://www.ncbi.nlm.nih.gov, accession number PRJNA1399109.

## Supplementary material

Supplementary Figure 1 Developmental regulation of differentially expressed genes (DEGs) within waxy and soft-waxy maize kernels and Kyoto Encyclopedia of Genes and Genomes (KEGG) pathway analysis. **(A)** Venn diagram showing the number of DEGs. **(B)** KEGG classification analysis. **(C)** KEGG enrichment analysis of the carbohydrate metabolism pathway. W, waxy maize; SW, soft-waxy maize.
