# Supplementary figures and images for: Comparative analysis of starch structure and multi-omics profiles reveals candidate pathways associated with a novel soft-waxy maize trait
Source: Front Plant Sci. 2026 Apr 10;17:1784081. doi: 10.3389/fpls.2026.1784081 (PMC13105884; doi:10.3389/fpls.2026.1784081)

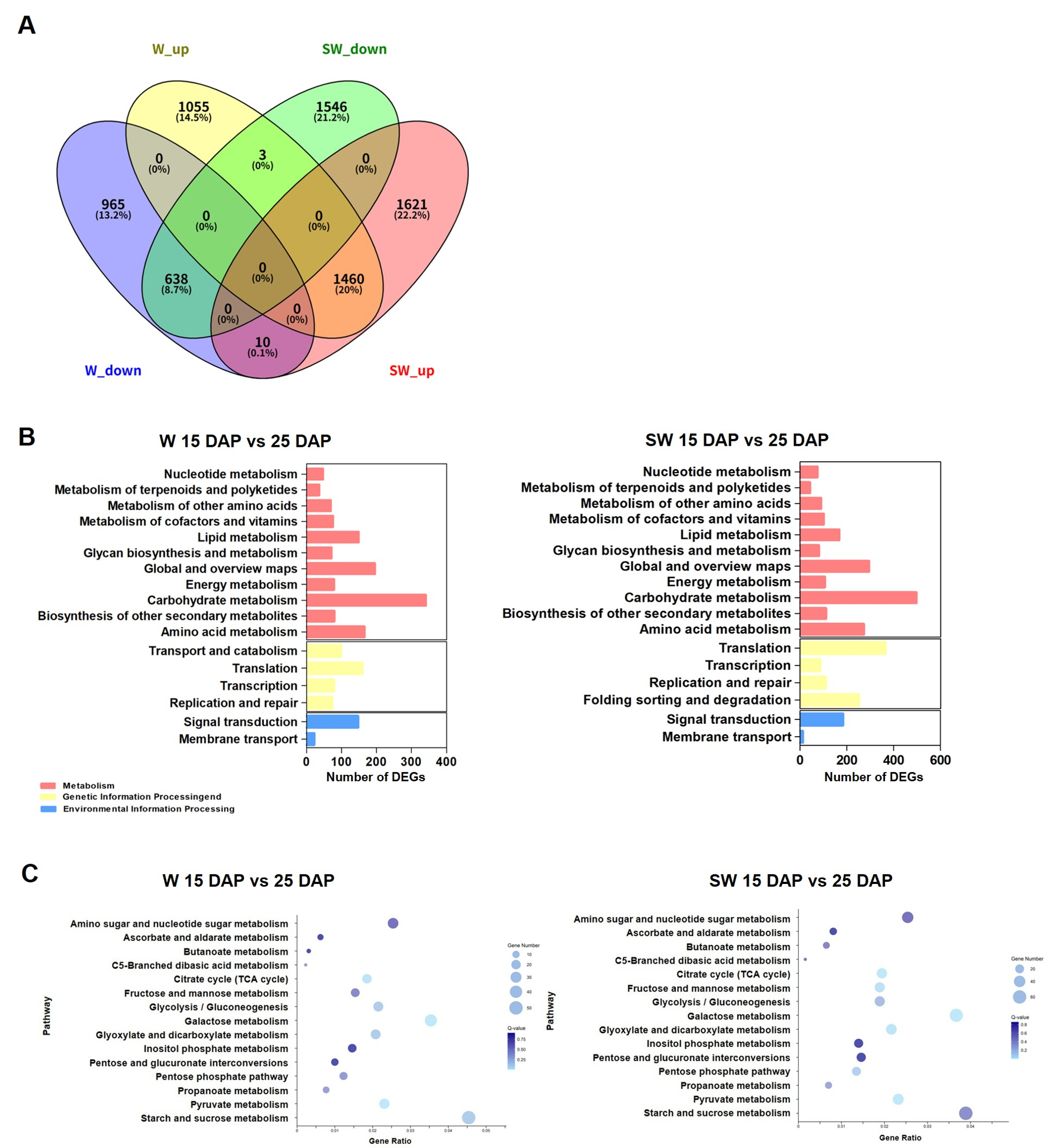

Supplement: Supplementary Figure 1 — Developmental regulation of differentially expressed genes (DEGs) within waxy and soft-waxy maize kernels and Kyoto Encyclopedia of Genes and Genomes (KEGG) pathway analysis. (A) Venn diagram showing the number of DEGs. (B) KEGG classification analysis. (C) KEGG enrichment analysis of the carbohydrate metabolism pathway. W, waxy maize; SW, soft-waxy maize. [file Image1.tif]
